# A Guideline for Effectively Synthesizing and Characterizing Magnetic Nanoparticles for Advancing Nanobiotechnology: A Review

**DOI:** 10.3390/s20092554

**Published:** 2020-04-30

**Authors:** Mohammad Reza Zamani Kouhpanji, Bethanie J. H. Stadler

**Affiliations:** 1 Department of Electrical and Computer Engineering, University of Minnesota, Minneapolis, MN 55455, USA; zaman022@umn.edu; 2 Department of Biomedical Engineering, University of Minnesota, Minneapolis, MN 55455, USA; 3 Department of Chemical Engineering and Materials Science, University of Minnesota, Minneapolis, MN 55455, USA

**Keywords:** magnetic nanoparticles, nanobiotechnology, nanomedicine, therapeutics, biosensing

## Abstract

The remarkable multimodal functionalities of magnetic nanoparticles, conferred by their size and morphology, are very important in resolving challenges slowing the progression of nanobiotechnology. The rapid and revolutionary expansion of magnetic nanoparticles in nanobiotechnology, especially in nanomedicine and therapeutics, demands an overview of the current state of the art for synthesizing and characterizing magnetic nanoparticles. In this review, we explain the synthesis routes for tailoring the size, morphology, composition, and magnetic properties of the magnetic nanoparticles. The pros and cons of the most popularly used characterization techniques for determining the aforementioned parameters, with particular focus on nanomedicine and biosensing applications, are discussed. Moreover, we provide numerous biomedical applications and highlight their challenges and requirements that must be met using the magnetic nanoparticles to achieve the most effective outcomes. Finally, we conclude this review by providing an insight towards resolving the persisting challenges and the future directions. This review should be an excellent source of information for beginners in this field who are looking for a groundbreaking start but they have been overwhelmed by the volume of literature.

## 1. Introduction

Advancement of nanotechnology has extensively expedited the emergence of novel magnetic nanostructures by reducing the dimensions to 2D nanomaterials, such as thin films and supperlattices, or 1D nanomaterials, such as magnetic nanowires (MNWs), and even 0D, such as spherical magnetic nanoparticles. The excellent quantum efficiency achieved using these nanomaterials has made them useful building blocks for diverse research areas, including medical treatment [1,2,3,4,5], environmental science [6,7], and quantum devices [8,9,10,11]. These magnetic nanostructures have opened numerous opportunities for scientists in different disciplines such as nanomedicine, molecular biology [12,13,14], applied physics, and nanostructured materials [15,16,17,18,19,20].

**Figure 1:**
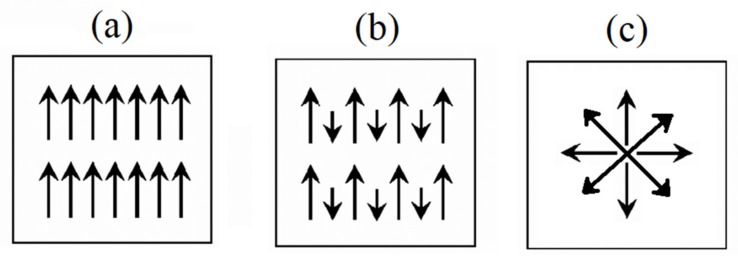
Schematic illustrating the dominant magnetic moment configurations in nanoparticles: (**a**) ferromagnetic: summing up long range ordering of magnetic moments, (**b**) ferrimagnetic: subtracting long range ordering of magnetic moments, opposite directions in the neighboring domains, and (**c**) superparamagnetic: continuous fluctuations of the magnetic moment leading to a net zero magnetic moment.

Among all magnetic nanostructures, the low dimension magnetic nanostructures, 0D and 1D magnetic nanoparticles, have attracted huge attention over the last few decades as they provide multimodal functionality priming multitude aspects of the nanomedicine and therapeutics applications. As the magnetic nanoparticles’ dimensions and size are reduced, due to the competition between the magnetic energies, in addition to their composition, the magnetic nanoparticles present different magnetic behaviors, such as ferromagnetic, superparamagnetic, and ferrimagnetic (see Figure 1). The ferromagnetic and superparamagnetic nanoparticles are opposite, as the former ones have long range ordered magnetic moment leading to have non-zero magnetization at zero fields, while the latter do not possess a stable magnetic moment, due to thermal fluctuations, leading in zero magnetization at zero fields. Note that ferrimagnetic nanoparticles are an intermediate state between these two states where they would be superparamagnetic if their sizes are sufficiently small so that no domain walls can be formed.

The multimodal functionality of the magnetic nanostructures requires an accurate and precise characterization of these nanostructures, which may inhibit or enhance their use depending on the application. Unfortunately, the high yielding nanofabrication processes for magnetic nanoparticles do not allow perfectly identical production, leading to variation in their magnetic characteristics and functionalities, ultimately inefficient for the proposed application. Consequently, in order to suppress this persistent challenge, it is important to understand the strengths and weaknesses of the synthesis processes as they are fundamental for producing identical magnetic nanoparticles with unique properties. Furthermore, understanding the reliability and validity ranges of the diverse characterization techniques is crucial for determining the most effective synthesis process for achieving magnetic nanoparticles with desired properties for a particular bio-application. In this review, we briefly provide details regarding the most commonly used synthesis processes to realize the most effective approach for tailoring magnetic nanoparticles. We then explain the diverse techniques used for characterizing the size, morphology, composition, and magnetic properties of these magnetic nanoparticles. Finally, we provide objective recommendations for selecting the most effective synthesis approach for producing magnetic nanoparticles for specific applications.

## 2. Synthesis Processes

Magnetic nanoparticles can be divided into two groups based on their dimensions: 0D and 1D. Each category can be further divided into sub-categories based on their shapes or aspect ratios, defined as the ratio of the longitudinal size to the lateral size. For example, 1D magnetic nanoparticles include nanodiscs, which are particles with aspect ratios equal or smaller than one and nanowires with aspect ratios larger than five. The magnetic properties of the magnetic nanoparticles determine the best synthesis path. For example, the 1D ferromagnetic magnetic nanoparticles are mainly fabricated using either the template-assisted method or template-free methods. In both categories, the flux of ions can be produced using several methods, such as chemical vapor deposition, physical vapor deposition, atomic layer deposition, laser pulse deposition, and electrochemical deposition. Except for electrochemical deposition, other techniques are not very common in the fabrication of the magnetic nanowires because they require high energy and vacuum pressure that are costly. Furthermore, in addition to the very low yields of these techniques, they also suffer from uniform growth of the magnetic nanowire, especially if high aspect-ratio magnetic nanoparticles over 1000, such as in template-assisted electrodeposition of magnetic nanowires, are desired. Therefore, here we focus on the electrochemical deposition technique that requires a template for synthesis. To date, numerous methods for synthesizing the magnetic nanoparticles have been proposed and successfully employed for the fabrication of diverse magnetic nanoparticles. Considering the cost and controllability of size/shape, all these synthetic methods can be categorized into two main categories according to the used solvent: aqueous or non-aqueous solvents [21]. The aqueous-based magnetic nanoparticles are relatively cheap; however, controlling their sizes and shapes is very challenging. The non-aqueous-based methods provide good control of the size and shape while they are relatively more expensive compared to the aqueous-based methods. Here, we provide a brief review of the most popular synthetic methods.

### 2.1. Co-Precipitation

**Figure 2:**
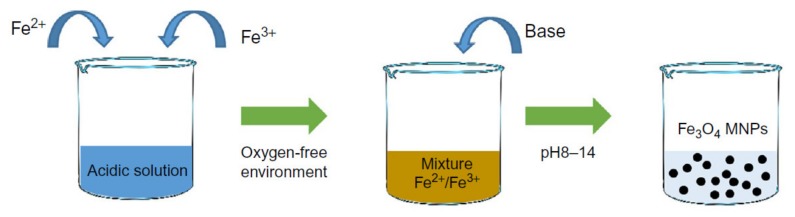
A schematic of the synthesis of iron oxide magnetic nanoparticles using the co-precipitation method [26]. In this example, the precursors (Fe^2+^/Fe^3+^ chlorides, sulfates, or nitrates) are dissolved in an acidic solution. Then, a strong base is added to increase the pH > 8 in a non-oxidizing environment.

Co-precipitation is the most commonly used approach that can be done at room temperature or elevated temperature (Figure 2). The solution consists of mixing ferrous and ferric ions in a molar ratio of 2:1 protected using an inert gas. In this method, the solution pH is a very important factor as a lower pH is desirable for nucleation of the Fe_3_O_4_ nucleus while the higher pH facilitates the growth of the Fe_3_O_4_ nucleus. The capability of this method for mass-production of magnetic nanoparticles has placed in a central position leading to several attempts to modify this method to enhance the magnetic nanoparticles’ magnetic properties and morphology [22]. For example, Wu et al. employed ultrasonic-assisted chemical co-precipitation to achieve magnetic nanoparticles with a nominal size of 15 nm with high purity [23]. Another example is the work by Pereira et al. where they synthesized magnetic nanoparticles with a nominal size of 5 nm using a one-step aqueous co-precipitation that employs alkanolamines [24]. These two examples represent a significant technological development as they are capable of mass producing magnetic nanoparticles with reduced average size while enhancing the magnetization moment. The size and shape control of the magnetic nanoparticles using this technique is very challenging, and furthermore, the presence of multi-phase magnetic nanoparticles is a common limitation [25]. The mass production of magnetic nanoparticles with large magnetization saturation usually suffers from particle aggregation. To overcome this limitation, a coating is essential, which was shown to readily be done using Ag and Au or introducing ligands.

### 2.2. High-Temperature Thermal Decomposition

**Figure 3:**
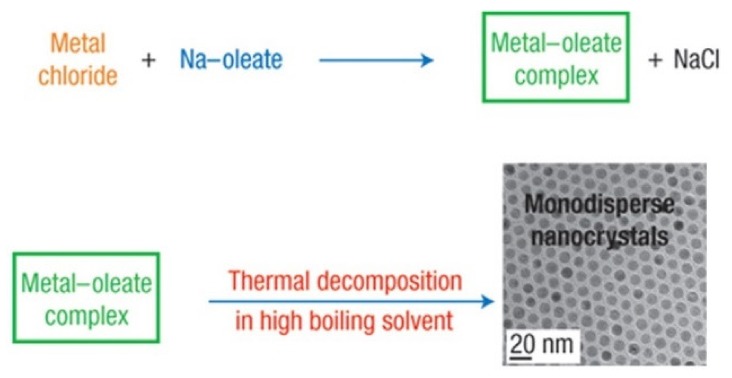
A schematic for magnetic nanoparticles preparation using thermal decomposition technique. Metal–oleate precursors were prepared from the reaction of metal chlorides and sodium oleate. The thermal decomposition of the metal–oleate precursors in the high boiling solvent produced monodisperse nanocrystals [30].

The thermal decomposition approach overcomes the size and morphology disparities limitation of the co-precipitation method. Generally speaking, the magnetic nanoparticles synthesized at higher temperatures provide more uniform size distributions [27]. The high-temperature decomposition also provides a route towards more crystalline magnetic nanoparticles (Figure 3). The main advantage of this method over co-precipitation is that it decouples the nucleation and growth of the magnetic nanoparticles leading to monodisperse, narrow size distribution, and highly crystalline magnetic nanoparticles [21]. It is possible to incorporate the inexpensive and non-toxic iron chloride to produce monodisperse magnetic nanoparticles without the need for size selection processes [28]. This approach also has the ability to control the crystallinity in ways suitable for producing various shapes, such as nano-cubic and nano-octahedral shapes [29]. Nevertheless, the magnetic nanoparticles synthesized using the thermal decomposition technique, especially those synthesized on aqueous media, tend to degrade in long term which makes their clinical applications debatable.

### 2.3. Hydrothermal and Solvothermal Synthesis

**Figure 4:**
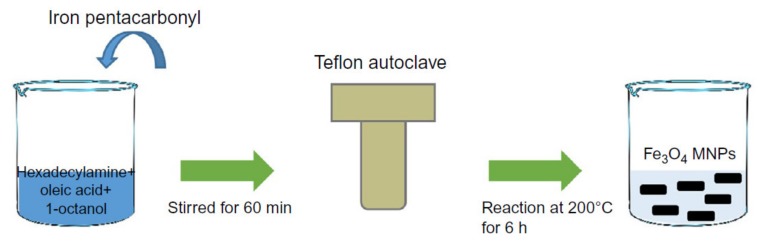
A schematic describing the hydrothermal synthesis approach. The process is similar to the co-precipitation; however, after preparing the mixture, the solution is transferred to an autoclave for further aging at high temperature and pressure for several hours [26].

Hydrothermal and solvothermal syntheses employ various wet-chemical techniques to form crystalline magnetic nanoparticles. Figure 4 shows a schematic of the hydrothermal method, where the process is carried out in high-pressure reactors or autoclaves to reach high pressures at high temperatures. This method uses either aqueous or non-aqueous solutions at high temperatures under high pressures to avoid the growth of dislocations in single crystal magnetic nanoparticles [30]. As a result, this method is suitable for the growth of crystalline phases that are unstable around their melting temperature. Furthermore, this method facilitates the growth of the magnetic nanoparticles that have a very high vapor pressure at their melting points while maintaining good control over the magnetic nanoparticles’ compositions [31]. This method is especially beneficial for the synthesis of hollow and controlled shape magnetic nanoparticles [32] including nanotubes and nanorings. It should be mentioned that this technique is very sensitive to the synthesis temperature as it can dramatically impact the reaction kinetics and nucleation rate [33].

### 2.4. Sol.-Gel and Polyol Methods

Sol-gel and polyol methods use essentially the same process but in a different direction, in which the sol-gel process is an oxidation reaction whereas the polyol process is a reduction reaction. The sol-gel synthetic approach is a well-known and widely used method in material science for the fabrication of metal oxides. This method usually starts with a colloidal solution acting as a precursor for either discrete nanoparticles or network polymers. Typically, a sol is a stable dispersion of colloidal nanoparticles or polymers in a solvent. Similarly, the gel could be either a colloidal gel, a network built from the agglomeration of colloidal nanoparticles, or a polymer gel, in which the nanoparticles have a polymeric sub-structure made by aggregation of sub-colloidal nanoparticles. Sol-gel processes usually are done at room temperature and the heat treatment can be done if high crystalline structures are desired [34]. The sol stage plays a critical role in the quality of the final nanoparticles made through this approach because the final size and saturation magnetization of the nanoparticles highly depends on the sol stage. The shape and crystallinity of the nanoparticles produced by this method are very sensitive to the type of precursors of the initial colloidal solution. As a result, this method is capable of producing nanospheres, hollow nanocages, and nanorods by controlling the water to acid ratio. Further adjustment on the temperature, pressure, and hydrous state can be done to tailor the phase of the nanoparticles [35].

In the polyol method, on the other hand, the polyols serve as both solvent and reduction agent and it applies stabilizers to prevent nanoparticles aggregation while controlling the growth of nanoparticles. The polyol method is done at high temperatures, the boiling temperature of the solution, but it does not require to be done at high pressure as it is required by the hydrothermal methods. This method can be done using different polyol solvents, for example, triethylene glycol (TREG), with high uniformity of morphology and colloidal stability nanoparticles. The magnetic nanoparticles produced by the sol-gel and polyol methods contain hydrophilic ligands on the surface that enhance their colloidal stability in the aqueous and non-aqueous solvent, an advantage compared to the magnetic nanoparticles produced by the co-precipitation method. Regardless of the high cost and safety issues associated with the sol-gel and polyol methods compared to the co-precipitation method, the sol-gel and polyol methods result in magnetic nanoparticles with significantly higher crystallinity and saturation magnetization [30].

### 2.5. Microemulsion Methods

Microemulsions are isotropic, stable, and clear mixtures of water, oil, and a surfactant [26]. The most commonly used microemulsion approaches for the synthesis of the magnetic nanoparticles are reverse, in which water dispersed in oil (w/o), and direct, in which oil dispersed in water (o/w) [21]. The surfactant could be a monolayer molecule with a hydrophilic tail dissolved in the water and a hydrophobic head dissolved in the oil, or vice versa.

**Figure 5:**
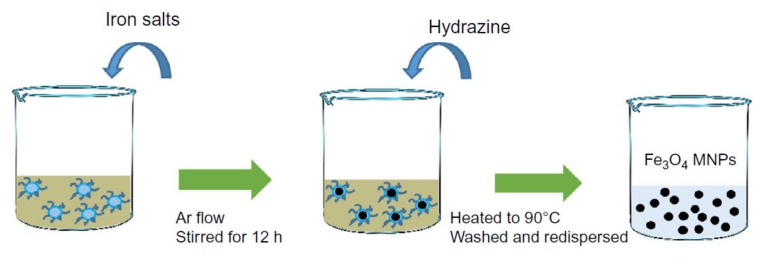
A schematic for the microemulsion process [26]. In this process, the iron (II) sulfate and iron (III) chloride salts are used in addition to hydrazine, which decreases the nanoparticles formation.

Figure 5 schematically shows the microemulsion method, where the blue circles (also known micelles) are the magnetic nanoparticles precursors surrounded by surfactant molecules. The initial concentration and form of the surfactants are the keys to the final size and growth of the magnetic nanoparticles. For example, Darbandi et al. reported highly uniform size distribution and crystalline magnetic nanoparticles using the microemulsion method at room temperature [36]. It was shown that the presence of the surfactant residuals on the magnetic nanoparticles provides high molecular bonding affinity that makes this method highly desirable for producing magnetic nanoparticles for the detection and purification of the proteins in a solution as well as delivering vitamins [37].

### 2.6. Sonolysis or Sonochemical Methods

**Figure 6:**
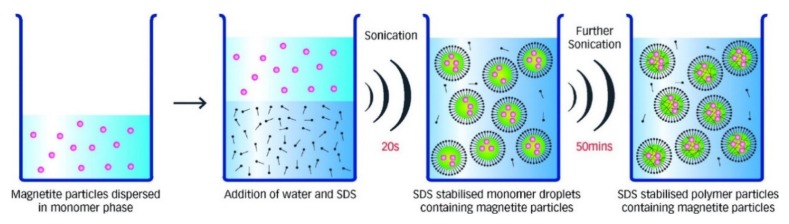
A schematic of the encapsulation of magnetic nanoparticles in latex nanoparticles using the sonochemically-driven miniemulsion polymerization technique [42]. Sodium dodecyl sulfate (SDS) is added to magnetite nanoparticles in the monomer phase followed by sonication to stabilize the surrounding magnetite nanoparticles.

Sonolysis or sonochemical methods employ high-intensity ultrasound irradiation to take advantage of the chemical effects induced by the acoustic cavitation for forming novel magnetic nanoparticle structures [38,39]. The ultrasonic irradiation creates bubbles that undergo continuous compression and expansion leading the oscillation of the bubbles (Figure 6). The oscillating bubbles accumulate the ultrasonic energy that continuously increases until causing the collapse and releasing the stored energy in the bubbles. Once the bubbles collapse, a highly localized energy burst is released that significantly increases the temperature and pressure at an extremely short time. In general, ultrasound-based irradiation is an excellent pathway for producing nanocomposites, such as dispersed magnetic nanoparticles in reduced graphene oxides or magnetic nanoparticle-loaded latex beads [40]. Even though the sonolysis or sonochemical method are promising for the fabrication of magnetic nanoparticles with desired sizes and excellent magnetic saturation properties, this method suffers from the dispersity and controllability of the magnetic nanoparticles’ shapes. Furthermore, the magnetic nanoparticles synthesized using this technique are usually amorphous, porous, and agglomerated [41].

### 2.7. Microwave-Assisted Synthesis

**Figure 7:**
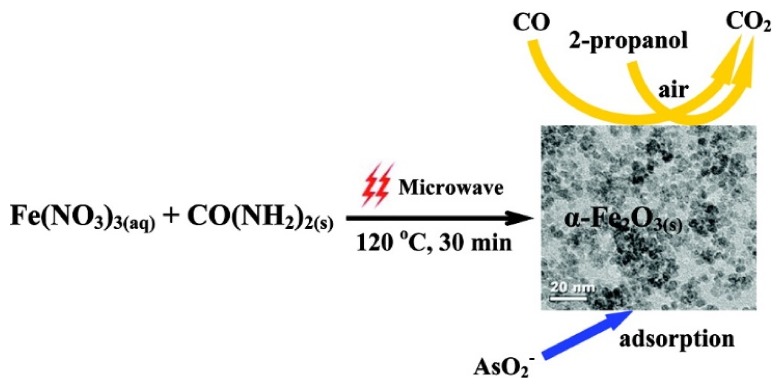
A schematic of magnetic nanoparticle preparation using the microwave-assisted technique at a temperature of 120 °C using a mixture of ferrite-nitrate and urea aqueous solution [47].

Microwave radiation forces molecules to reorient and oscillate with the electric field of the microwave signal (Figure 7). The strong oscillation at microwave frequencies results in intense internal heating that not only reduces the synthesis time but also significantly reduces the costs for nucleation and growth of the resulting magnetic nanoparticles [43,44]. The homogeneous excitation of the molecules using microwave signals have made this approach a strong tool for preparing the magnetic nanoparticles with controllable shape and size. One of the main advantages of the microwave-assisted synthesis is that this method can produce magnetic nanoparticles with different phases with an instantaneous coating that are desired for many applications, such as biomedical applications [30]. Furthermore, the microwave-assisted method is capable of producing magnetic nanoparticles with high colloidal stability that can be readily dispersed in water without any costly and complicated procedures for purification and ligand exchange [45]. These capabilities have made the microwave-assisted synthesis competitive to the thermal decomposition method for the mass production of magnetic nanoparticles. This method has been used for synthesizing magnetic nanoparticles as small as 6 nm up to 1000 nm and saturation magnetization comparable to the bulk values, where the crystallinity enhances with the increasing temperature of microwave heating [46].

### 2.8. Electrochemical Deposition

**Figure 8:**
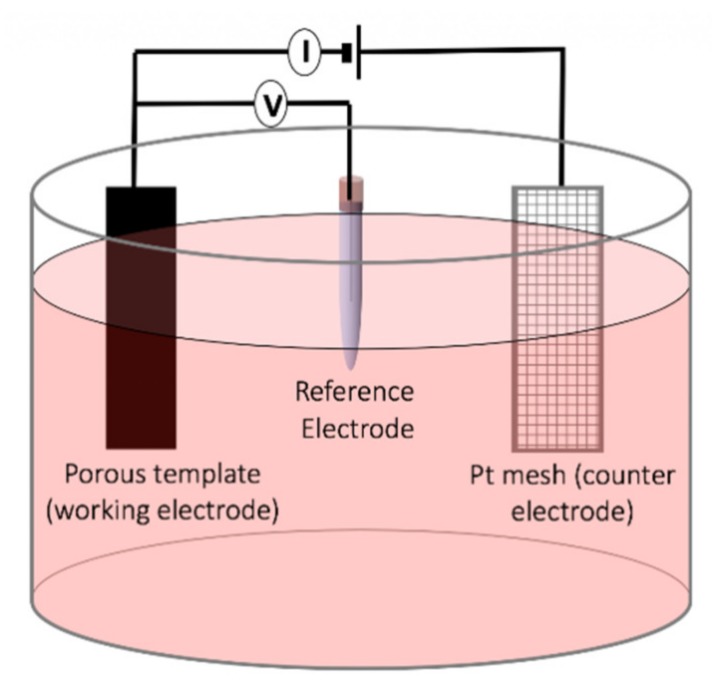
A schematic showing the synthesis of magnetic nanowires using a template-assisted electrodeposition technique [54]. After making the electrical contacts, the template is placed at anode and a platinum mesh is used as counter-electrode. The deposition current and voltage are controlled using the reference electrode, where the electric field between the working and counter electrodes forces the free ions to be deposited in the pores.

The template-assisted electrochemical deposition has been broadly used in the synthesis of the magnetic nanowires as this method provides a highly controllable route for achieving precise dimension and compositions [48] (Figure 8). The template-assisted method can be divided into two categories depending on the template utilized, polymeric templates or anodic templates. The most commonly used polymer for synthesizing magnetic nanowires is polycarbonate because of its cost-effective approach, biocompatibility, and hydrophilic properties achieved by coating the polycarbonate templates. The hydrophilic property is the key for the fabrication of uniform and high aspect-ratio magnetic nanowires [49]. The polycarbonate templates are produced by ion irradiation of the row polycarbonate temples followed by a chemical etching process for opening the pores, which determines the final pore diameter. Due to the randomness of the ion irradiation, the distribution of the nanopores in polycarbonate templates is non-uniform. This features of polycarbonate templates have been used to synthesize interconnected networks of magnetic nanowires [50]. The aluminum anodic oxide templates, on the other hand, are relatively more expensive compared to the polycarbonate templates but they provide magnetic nanowires with very uniform diameters. The anodic aluminum oxides are prepared using both one-step anodization and two-step oxidation process after patterning an aluminum foil, where the two-step anodization leads to a significantly uniform distribution of the nanoporous [51,52]. A distinct advantage of anodic aluminum templates is that they provide flexibility to engineer the diameter along the porous leading to fabricate multi-diameters or tapper magnetic nanowires [53]. Particularly, electrochemical deposition is a strong tool for the synthesis of the magnetic nanowires as multi-segmented and/or multi-component, such as alloys.

It should be mentioned that electrochemical deposition has been also used for synthesizing non-cylindrical magnetic nanoparticles, such as nanospheres [55]. Despite the high costs associated with the electrochemical synthetic approach, this method is very useful compared to other methods if very high purity products with controlled size and shape are demanded. The electric field distribution within the electrodes plays a critical role in the magnetic nanoparticle size and aggregation during the synthesis. A challenge during the synthesis of magnetic nanoparticles using the electrochemical methods is the formation of the metallic Fe. To overcome this challenge, one should apply a more anodic potential over a long time, which makes this method unfavorable compared to the other synthetic methods [56] for mass-production.

### 2.9. Biosynthesis Methods

The biosynthesis method employs a microbial enzyme or a plant phytochemical with reducing properties that make this method eco-friendly. Traditionally, magnetotactic bacteria and iron-reducing bacteria are used to synthesize magnetic nanoparticles [57]. This method was shown promise for doping iron oxide magnetic nanoparticles with cobalt with improved magnetization properties [58]. More recently, several attempts have been put forth to employ human stem cells to synthesize re-magnetized magnetic nanoparticles [59]. The type of the bacteria and the synthesis conditions (aerobic or anaerobic), determine the phase of the resulting magnetic nanoparticles. For example, maghemite magnetic nanoparticles with superparamagnetic characteristics can be produced using *Actinobacter* bacteria under aerobic conditions [60]. In general, the mechanism of the biosynthesis of magnetic nanoparticles has not been well understood in order to clarify the controlling parameters of the shape and sizes while maintaining the desired saturation magnetization of the magnetic nanoparticles [61].

### 2.10. Other Techniques

In addition to the aforementioned syntheses that are chemical approaches, magnetic nanoparticles have been also produced using physical methods. The physical techniques are in their majority top-down processes where a bulk magnetic material is decomposed into magnetic nanoparticles. A few well-known examples of physical techniques are mechanical milling [26], electrical explosion of wires [62], and laser target evaporation [63]. Even though the physical approaches have higher production yield, up to 200 g/hr, they form almost 10% of magnetic nanoparticles for diverse applications. This is because the physical approaches have relatively high power consumption and controlling the shape and size distributions is difficult. 

## 3. Different Shapes

### 3.1. Sphere, Cubic, Octahedral, and Rhombohedral

The final morphology of the magnetic nanoparticles not only depends on the synthetic method used, but also on the solution composition, temperature, and pressure [30]. For example, the precipitation of an iron (II) salt in alkaline media in the presence of a mild oxidant such as potassium nitrate results in magnetic nanocubes [64] rather than nanospheres. This is because the aforementioned parameters accelerate crystal growth over specific facets while hindering the growth over other facets by slowing it down. Aside from the aforementioned parameters, the ratio of the compounds in the solution is the most critical parameter determining the shape of the magnetic nanoparticles. For example, when the Fe (II):OH^−^ ratio gets close to 0.77, the achieved magnetic nanoparticles are cubic, while the shape changes to octahedral as the ratio approaches 1.65. It should be noted that the crystal growth at different facets is also controllable by precise adjusting the pH during the synthesis. At low pH, the OH^−^ concentration is negligible thus the growth takes place mainly by aggregation, where the growth kinetics are much faster. In another words, the primary nanoparticles are not repelling each other because they are not sufficiently charged and the aggregation is followed by subsequent recrystallization leading to octahedral nanoparticles with a broad range of sizes, from a few nanometers to micrometers [65,66]. 

As mentioned, thermal decomposition is able to differentiate the nucleation and growth process during the synthesis. By changing the precursors, it is possible to obtain octahedral magnetic nanoparticles using a thermal decomposition method. For example, the decomposition of iron (II) oleate in tetracosane in the presence of oleylamine results in octahedral nanoparticles nucleated by the selective binding of oleylamine to {111} facets [67]. Interestingly, the heating rate of the solution also was found to lead to octahedral nanoparticles. A synthetic strategy laying between the aqueous and organic media consists of the hydrolysis of Fe (II) acetate in the presence of oleylamine dissolved in xylene. Heating the reaction mixture followed by a fast injection of water triggers the hydrolysis of the Fe-oleylamine complex leading to nanocubes as well.

Recently, a three-step approach was reported for the synthesis of the rhombohedral magnetic nanoparticles [68] through a three-step synthetic approach: (1) synthesis of antiferromagnetic nanoparticles, (2) nanoparticle coating, and (3) subsequent reduction of the core material to magnetite. This approach comprises the generation of hematite nanoparticles, further encapsulation in silica and final reduction to magnetite, in which the first step is crucial for the growth of rhombohedral scaffolding and it is achievable by solvothermal synthesis.

### 3.2. Nanodiscs

The easiest synthetic approach for the fabrication of the nanodiscs is the precise control of the deposited charge during the electrochemical deposition. Since the length, or thickness in the case of nanodiscs, can be accurately controlled using the applied potential and deposition time, the electrochemical deposition is the strongest tool for achieving this morphology. However, if nanodiscs of a very small size are desired, this technique becomes costly compared to the other synthetic approaches, even for small volumes of magnetic nanoparticles. Alternatively, both one-step and two-step synthetic approaches, such as the solvothermal technique, become a good alternative [69]. For the one-step synthetic techniques, a promising approach must delay the nucleation of the nanoparticles. The nucleation can be delayed in the absence of the water during the formation of the common nanoparticles, such as nanospheres [65]. In the aforementioned aquatic-based synthesis techniques, water acts as an accelerating agent, which increases of the thickness while suppressing the diameter growth. Reducing the size of nanodiscs while maintaining the aspect ratio is also possible through two-step synthesis approaches [70]. Moreover, it was reported that hematite nanodiscs can be achieved by the hydrolysis of iron (II) chloride in a mixture of water/ethanol in the presence of sodium acetate [71]. In this approach, the diameter and thickness of the hematite nanoparticles can be controlled by the amount of water in the solvent and sodium acetate.

### 3.3. Elongated Nanoparticles

The elongated nanoparticles are the results of hindering the crystal growth rate at a specific direction while accelerating the growth rate at other facets. This can be done by incorporating precursors during the synthesis of the template-free synthetic approaches or simply using a template during the synthesis. The elongated nanoparticles are technically the 1D magnetic nanoparticles which are described in the literature with different names such as nanowhiskers [72] or nanorods [73], nanorices [74], nanobelts [75], nanospindles [76], and nanowires [77]. The different terms are attributed to their final morphology, the geometry of the edges, and the axial ratios of the lateral dimension to the longitudinal dimension. Recently, numerous attempts were taken place to achieve a one-step direct synthesis of elongated nanoparticles as opposed to the conventional methods as they require templates for controlling the morphology. Comparatively, the final properties, such as crystallinity, of the elongated nanoparticles highly depend on the synthetic approach used [65]. The template-free synthetic approaches are mainly limited to magnetic nanoparticles with small aspect-ratios [78], on the order of 3–10. For example, the hydrolysis and oxidation of the iron (II) sulphate in water in the presence of carbonate ions was shown to result in magnetic nanoparticles with an aspect ratio of 3 to 4 at room temperature [74]. In this approach, the concentration of the iron (II) sulphate and carbonate, air flow rate and reaction time, additive precursors, and pressure are all influencing factors.

The strongest synthetic approach for elongated nanoparticles, especially with a broad range of aspect ratios in order of 1 to 1 × 10^4^, is template-assisted electrochemical deposition [77]. By adjusting the potential and deposition time, the aspect ratio can be easily controlled. The final aspect ratio depends on the ratio of the template pore diameter to thickness. As mentioned, the main advantage of the electrochemical deposition is that it can synthesize multi-compounds and multi-segmented elongated nanoparticles. The length uniformity can be controlled by applying step potentials, additive precursors, and the solution temperature.

## 4. Characterization

Similar to other types of nanoparticle, precisely characterizing of the magnetic nanoparticles is very important as it determines the reproducibility of the results. Characterization of magnetic nanoparticles in terms of their size, shape and composition is particularly substantial as their magnetic properties are significantly influenced by those parameters. For example, if the size of a magnetic nanoparticle shrinks sufficiently such that it no longer can hold a domain wall, the coherence between the spins results to superb magnetic properties. Similarly, the shape of the magnetic nanoparticles causes magnetic inhomogeneity leading to quantum effects that cannot be achieved in the bulk states. In nanobiotechnologies, particularly in biosensing, the accurate characterization of the composition and surface coating is vital because their biocompatibility and sensitivity are highly relied on these characterizations. In this section, we briefly explain the highly characterization techniques that have been utilized to demonstrate their functionalities in diverse applications, from quantum storage to nanomedicine.

### 4.1. Shape, Size, and Composition

#### 4.1.1. Transmission Electron Microscopy (TEM)

TEM is a microscopy technique that exploits the interaction between a uniform flux of electrons and the nanoparticles under study. The interaction of electron flux leads to a part of it being transmitted through the nanoparticles while the rest are scattered, where the interaction depends on the size, shape, and elemental composition of the nanoparticles. TEM is the most popular technique to measure nanoparticles’ shape, size, and homogeneity because it provides direct images of the nanoparticles. Recent advances in TEM imaging, such as liquid-phase TEM, not only directly characterizes magnetic nanoparticles size and morphology but also characterizes the interparticle distances in a solution [79,80], which was shown to be a critical parameter for the magnetic response of the magnetic nanoparticles. Interestingly, TEM facilitates the real-time imaging for demonstrating the dynamic transformation in nanoparticles over time [81]. For example, TEM was used to visualize the biodegradation of the coating of nanoparticles in the presence of biological entities, such as bacteria. It also was found to be a very strong approach for real-time monitoring of the dynamic growth of the magnetic nanoparticles in suspension [82]. Nevertheless, TEM is very costly and slow for the characterization of nanoparticle assemblies if there are a large number of them with polydispersity. Furthermore, due to high absorption of the electron energy with liquid molecules, this technique is very tedious to measure the size and shape of the magnetic nanoparticles in suspensions. In these cases, the other methods, such as nanoparticle tracking analyzer (NTA) or dynamic light scattering (DLS), are more efficient [83].

#### 4.1.2. Dynamic Light Scattering (DLS)

**Figure 9:**
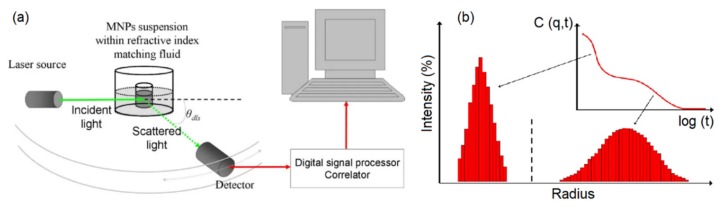
A schematic of the dynamic light scattering (DLS) setup [84] (**a**) and the acquired results (**b**). A laser light hits the magnetic nanoparticles in a continuous flow while the scatted light is monitored using a detector. The results are size distributions where the accumulative function can be calculated by taking an integral from the size distribution.

Dynamic light scattering (DLS) is a commonly used technique to find the size of particles suspended in colloidal solutions (Figure 9). DLS uses the Stoke-Einstein law to relate the light scattered from the nanoparticles in a colloidal solution to their hydrodynamic diameter. It is beneficial to have solutions with low concentration to avoid simultaneous multiple scattering events in order to achieve a more accurate analysis. The DLS technique has been used to realize the influence of the nanoparticle shapes, size, concentration, and surface coating on the colloidal stability of the magnetic nanoparticles [83]. For middle-size magnetic nanoparticles, the DLS technique provides accurate results for determining the size that was shown to match with the results derived from TEM and SEM images. However, for small size magnetic nanoparticles, the DLS technique does not match the TEM results due to the radius of curvature effects. The DLS is not a good technique for analyzing the magnetic nanoparticles if the heterogeneity and poly-disparities are high [84]. That is because the larger nanoparticles scatter substantially more light obscuring the detection of the scattered lights from the small nanoparticles. Furthermore, the DLS requires transformative analysis with several assumptions, especially when the nanoparticles are non-spherical or the polydisparity is high, which diminishes its accuracy [81].

#### 4.1.3. Nanoparticles Tracking Analyzer (NTA)

**Figure 10:**
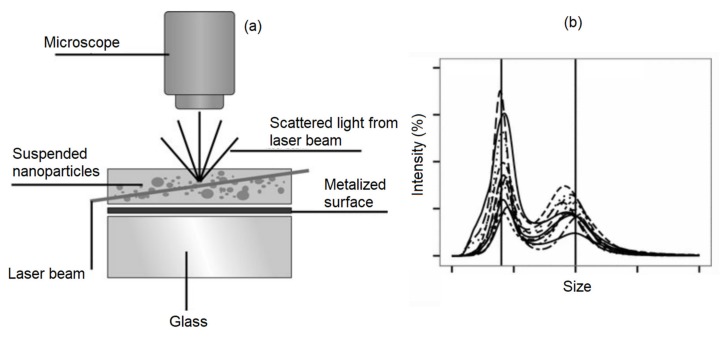
A schematic of the nanoparticles tracking analyzer [85]. (**a**) NTA employs a high speed camera to capture the light scattered by the Brownian motion of nanoparticles to determine the nanoparticles size distributions, and (**b**) is an example for a sample including two size distributions.

NTA takes advantage of both the light scattering and Brownian motion properties of the nanoparticles in a solution to determine the size distribution at a lower concentration limit compared to the DLS technique [85] (Figure 10). The main advantage of the NTA over DLS is that its results are not biased by the aggregation of larger nanoparticles. As a result, the literature shows that NTA provides more accurate results for both monodisperse and polydisperse samples compared to DLS [86]. The main difference between the NTA and DLS is that NTA tracks single nanoparticles while DLS analyzes an ensemble of nanoparticles with a high bias towards the larger nanoparticles. This different operation mode causes the NTA to be relatively slower with a more complex operation procedure than DLS. However, since NTA is also capable of preedictingt the magnetic nanoparticle concentrations [87], it has received a huge amount of attention over the last few years.

#### 4.1.4. X-ray Diffraction

Undoubtedly, X-ray diffraction is one of the key tools that has been extensively used to characterize both magnetic and non-magnetic nanoparticles. The X-ray diffraction technique provides detailed information regarding the phases, lattice parameters, crystalline grain size, and crystalline structures [81]. The composition, crystal structures and the nature of the phases can be determined by comparing the position and intensity of the X-ray peaks with the available reference database while the crystalline grain size can be determined using the broadening of the peaks [88]. Aside from the amorphous nanoparticles, the broadening of the X-ray peaks is mainly due to the nanoparticle/crystalline size and lattice strains. Practically, if the magnetic nanoparticles are big enough to hold more than one crystal boundary, the X-ray cannot distinguish between the boundaries leading in the misrepresentation of the crystalline grain sizes [89].

Numerous modifications have been applied to the X-ray diffraction to enhance its capability beyond demonstrating the chemical composition and/or crystallinity. For example, X-ray absorption includes both extended absorption fine structure and X-ray absorption near edge structure capable to measure the surface binding energy and density of states [90]. Another example is X-ray photoelectron spectroscopy that has been widely used for surface chemical analysis, electronic structures, elemental composition and oxidation state of the elements [90].

#### 4.1.5. Fourier Transform Infrared Spectroscopy

**Figure 11:**
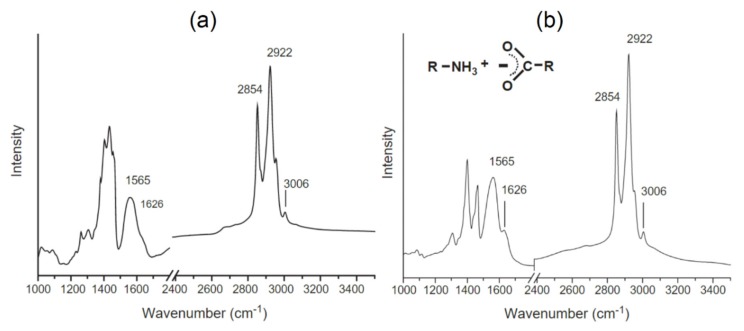
Comparing the FTIR spectra of (**a**) modified magnetic nanoparticles, (**b**) the modification composition [92].

Fourier transform infrared spectroscopy (FTIR) is another technique for determining the structure, size, and composition of magnetic nanoparticles. FTIR measures the absorption of electromagnetic radiation with wavelengths within the mid-infrared region. By comparing the FTIR spectra of pure magnetic nanoparticles or their modified versions, such as after adding a surface coating, one can quantify the composition (Figure 11). Once a molecule is excited with IR radiation, its dipole moment gets aligned and oscillates accordingly. Thus, the recorded spectrum gives the position of the bands related to the strength and the nature of the bonds providing information regarding the molecular structure and inter-molecule interactions [91]. FTIR is usually combined with differential electrochemical mass spectroscopy to further detect the volatile reactants. For example, Shukla et al. employed this method to illustrate the surfactant bonding on iron platinum (FePt) nanoparticles stabilized in a non-polar solution [92]. They showed that FTIR is capable of detecting the types of the bonding, either monodentate or bidentate, on the FePt magnetic nanoparticles. The relative low cost and high throughput of this technique have resulted in several attempts to push its limit to analyze magnetic nanoparticles of a few nanometers in size. For example, it was shown that FTIR can determine the crystallinity of magnetic nanoparticles with the average size below 15 nm [93].

#### 4.1.6. Nuclear Magnetic Resonance (NMR) Spectroscopy

NMR spectroscopy is an analytical method for the quantitative determination of magnetic nanoparticle structures. NMR spectroscopy is based on magnetic resonance of the nanoparticles’ nucleii under a strong magnetic field that induces an energy difference between the down and up spins. Even though NMR spectroscopy is a useful method for studying superparamagnetic nanoparticles, it fails to characterize ferromagnetic nanoparticles, such as nickel or cobalt magnetic nanowires, because their strong magnetization can cause a reduction of the relaxation time while drastically shifting the signal frequency and local magnetic field [94]. The NMR method has been used for characterizing the surface coating of the magnetic nanoparticle as it is very sensitive to electronic structures and molecular bonding on the surfaces [95] if they are sufficient to be detected. Practically, the NMR technique has sensitivity on the order of a few nanogram that can be enhanced to a few picograms [96].

#### 4.1.7. Mass Spectroscopy (MS)

Mass spectroscopy (MS) is known as a powerful and reliable tool for the analytical characterization of magnetic nanoparticles. The common MS tools have a sensitivity that is on the order of a few picograms, which is better than the conventional NMR technique [97]. This technique not only provides elemental information regarding the composition, chemical states, and structures of the magnetic nanoparticles but also quantifies the surface bioconjugations for targeting biomolecules [81]. Aside from the simplicity and universality of MS, it is a highly sensitive technique that can be coupled with separation techniques for real-time sorting applications. For example, the inductively coupled plasma MS (ICP-MS) is a robust, highly sensitive technique with a wide dynamic range for elemental analysis of the magnetic nanoparticles [98]. Another example is the single-particle operation mode ICP-MS those improvements on the identification of the concentration and size distribution of magnetic nanoparticles [99].

#### 4.1.8. Thermal Gravimetric Analysis (TGA)

Even though FTIR provides information regarding the presence of binding on the surface and its type, it does not provide volumetric information, such as mass to mass ratio of the magnetic nanoparticles to its coating. This limitation can be addressed using thermal gravimetric analysis (TGA), which provides information about the composition and mass of the coating [100,101,102]. This technique increases the sample temperature while monitoring the mass change of the sample as it changes due to the degradation of components. As a result, this technique is not only capable to quantitatively determine the mass of the coating of the magnetic nanoparticles but also determines the compositional purity and the thermal stability of the coating [103]. For example, Ziegler-Borowska et al. conducted TGA experiments in both air and nitrogen where they showed the modified chitosan has low thermal stability; however, once this coating thermally degrades, its degradation products form a stable surface layer [104]. The drawback of this method is its destructive analysis and the minimum initial nanoparticle mass [81].

### 4.2. Magnetic Characterization

#### 4.2.1. Hysteresis Loop Measurement

As the size of the magnetic materials decreases towards the nanoscale, they exhibit substantially different magnetic properties compared to their bulk state. This change in the magnetic properties is due to two facts. First, the magnetic nanoparticles can no longer hold multiple domains to balance their magnetiostatic energy and exchange energy [105]. Second, the number of the atoms on the surface becomes comparable to the number of atoms in the volume. The former means that they behave as a single domain nanoparticle, where they behave like superparamagnetic if the thermal fluctuations become significant compared to the energy barrier. The latter means that, at a few nanometer size, the unpaired electrons become more dominant leading to additional anisotropies, such as surface anisotropy. The major hysteresis loop measurement is the key technique to measure the basic hysteretic information of any magnetic nanoparticle. The hysteresis loops can be measured using a superconducting quantum interface device (SQUID) or vibrating sample magnetometry (VSM), in which the SQUID has a significantly higher resolution. The major hysteresis loop provides the saturation magnetization, remanence magnetization, and the coercivity. These parameters are sufficient to describe the magnetic response of isolated-single domain magnetic nanoparticles, or those with negligible magnetic interactions, with respect to a magnetic field. However, if further characterization is required or the interaction fields among the magnetic nanoparticles are not negligible, more advanced magnetic characterization techniques are needed [106,107,108]. Importantly, the hysteresis loops are unable to discriminate between the different phases if there are more than one in magnetic nanoparticle assemblies.

#### 4.2.2. Mössbauer Spectroscopy

**Figure 12:**
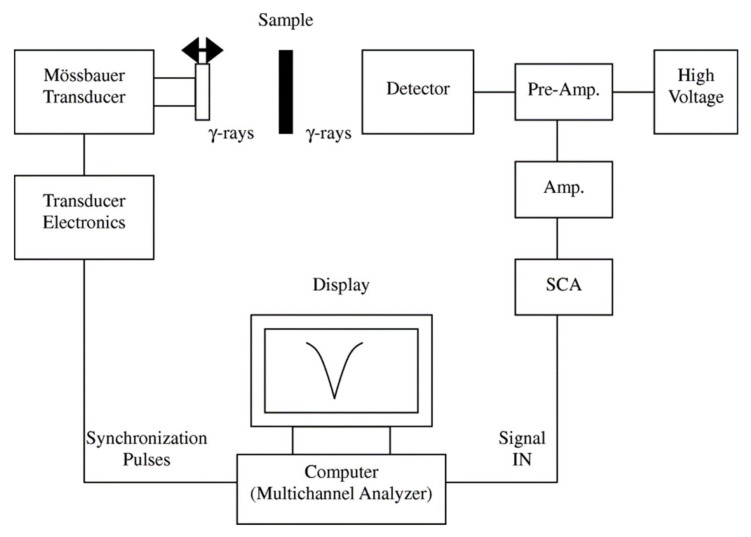
A schematic of the Mossbauer spectroscopy setup [111].

Mössbauer spectroscopy (Figure 12), is a powerful analytical technique to evaluate the oxidation state, spin states, and spin ordering of Mössbauer-active elements (such as Fe) for the identification of magnetic phases [109,110]. Mössbauer spectroscopy can also provide insight into the quantification of the thermal blocking/unblocking (superparamagnetic response) and magnetic anisotropy energy if the measurements are conducted as a function of temperature. The Mössbauer spectroscopy shift is an important parameter that arises from the nuclear-energy shift that is caused by the Coulombic interaction between the nucleus and the electron density at the site of the nucleus [81]. As an example, Oh et al. used Mössbauer spectra as a probe to quantitatively study the local state of electrons on the surface of the FeCo nanoparticles [111]. They investigated the process parameters on the magnetic properties of the FeCo nanoparticles using Mössbauer spectroscopy. Furthermore, Lange and et al. showed that Mössbauer spectra can be used to determine the hyperfine interactions between nuclei and their surroundings because this method is very sensitive to the local structural and chemical environment of the Mössbauer-active elements [112]. In this direction, Tiano and co-workers utilized Mössbauer spectroscopy and SQUID to draw the relation between the magnetic properties and the composition of several magnetic nanoparticles, such as Mg, Fe, Co, and Ni [113]. The Mössbauer isomer shift is not technically a probe for determining the oxidation number of the dopant atoms because it only determines the charge state on the nucleus [114]. Therefore, if both Fe^2+^ and Fe^3+^ species persist in a Fe-doped nanoparticles, the electron redistribution from the dopant sites to the crystal matrix leads a very similar shifts for both species.

#### 4.2.3. Ferromagnetic Resonance (FMR)

FMR is a spectroscopic technique probing the nanoparticle magnetization induced by the magnetic moments of the dipolar coupled of unpaired electrons. The width of the FMR peak was shown to be related to the size, shape, defects density, and surface anisotropy. For example, Diehl et al. conducted FMR spectroscopy experiments on both crystalline and imperfect magnetic nanoparticles revealing that this technique is able to illustrate the coherence of the lattice drastically impacts the magnetic inhomogeneity and anisotropic properties of nanoparticles [115]. The same authors also reported that surface defects can be detected at low temperatures using FMR. It was shown that decreasing the temperature or increasing the magnetic nanoparticle size causes a shift in the resonance field leading to enhancement of the FMR peak asymmetry and broadening [115]. It should be mentioned that FMR usually predicts the size of the nanoparticles smaller than the actual values measured by TEM due to the presence of the magnetically disordered layer on the surface.

#### 4.2.4. Magnetic Susceptibility

Magnetic susceptibility is defined as the ratio of the magnetization to the applied field indicating how strongly whether a nanoparticle is repelled by or attracted into a magnetic field. This technique is able to quantify the magnetic nanoparticles coated with polymeric or organic/inorganic materials. Consequently, this technique has been used to determine the mass of the surface coatings to the magnetic mass of nanoparticles [116,117]. Magnetic susceptibility also has been used to acquire the size and size distribution of magnetic nanoparticles, and a good agreement was observed by comparing to the TEM results [117]. The field-dependent magnetic susceptibility was shown to provide information regarding the magnetocrystalline anisotropy of the magnetic nanoparticles. Due to the limited dynamic range of the susceptometers, this technique most often used over a range of temperature rather than frequencies that provides insight into the heating efficiency of the magnetic nanoparticles for certain applications.

#### 4.2.5. Electron Holography

**Figure 13:**
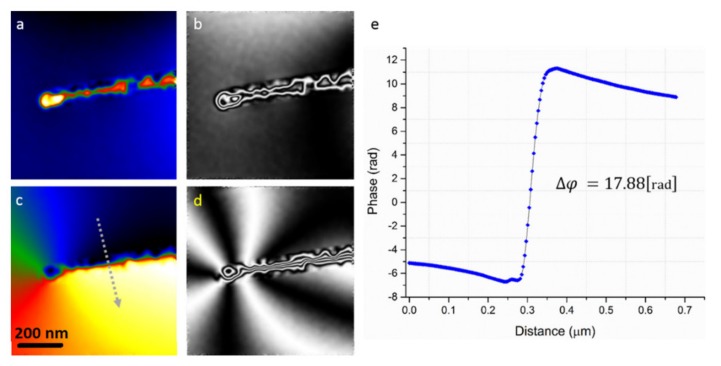
An exemplary experimental result showing magnetic nanowires captured using the electron holography technique [120]. (**a**,**b**) are the electrostatic phase and its amplification, respectively. (**c**,**d**) are the magnetic phase and its amplification. (**e**) is the phase shift across the arrow in (**c**).

Electron holography is a holographic technique using electron waves for imaging. This technique uses a high spatial and temporal coherence of an electron beam to acquire holographic imagery. The main advantage of electron holography is that this technique provides direct information regarding the internal magnetic structures rather than via the stray fields or only the surface magnetization states (Figure 13). Among the several different holography configurations, the off-axis and in-line configurations are the most commonly used techniques in imaging nanoparticles. For example, Ortega et al. reported the off-axis electron holography under Lorentz microscopy conditions to observe the magnetization distribution and to determine the saturation magnetization of multi-segmented FeGa/Cu nanowires [118]. They showed the presence of an antiferromagnetic configuration along the FeGa/Cu nanowires even though the magnetic nanowires are ferromagnetically coupled. Electron holography was also shown to be useful for visualizing the magnetic domain walls and/or domain wall pinning in magnetic nanoparticles with high resolution compared to other magnetic imaging techniques, such as magnetic force microscopy. For example, Biziere et al. reported on observation of the magnetic domain walls in magnetic nanowires leading to illustrating the presence of two types of domain walls transferring to each other by manipulating the diameter [119].

#### 4.2.6. Remanence Curves Technique

The remanence curves technique has been widely used to qualitatively determine the nature of the interaction fields among magnetic nanoparticles in assemblies. The remanence curves technique characterizes magnetic nanoparticles by subtracting isothermal remanence and DC demagnetization curves and comparing the results with the Stoner-Wohlfarth model, also known as Henkel plots. The isothermal remanence measures the remanence by applying and removing an ascending field to initially demagnetized nanoparticles [121], while the DC demagnetization measures the remanence by applying and removing a descending field to initially saturated nanoparticles [122]. For non-interacting nanoparticles, both curves are identical, meaning there is no interaction. However, the curves deviate for interacting nanoparticles, where the sign and strength of the deviation qualitatively determine the interaction fields. Recently, several attempts have been made to use this technique for quantitative analysis of the interaction fields [123,124]. For example, Huerta et al. used it to study the interaction fields between arrays of magnetic nanowires where the authors showed interaction fields are dominated by dipole-fluctuation effects [123]. In their approach, they used the difference between the field where the DC demagnetization curve is zero and isothermal remanence curve is 0.5 to quantify the interaction fields. In another example, Moya et al. used the remanence curves technique combined with the ZFC/FC to quantify the interaction fields [125].

This technique also has been proposed to probe the spin disorder in magnetic nanoparticles [126]. For example, Toro et al. reported that the dips in the DeltaM plots, calculated by subtracting the remanence curves, are not necessarily due to the interaction fields as they could be due to spin disorder, an inhomogeneity of spin distributions in the magnetic nanoparticles [127]. Despite the simplicity and straightforward measurements and analysis of the remanence curves technique, this technique is limited to magnetic nanoparticles having strong magnetic anisotropy, in which their coercivity is larger than the interaction fields [128]. As a result, it is unable to determine the interaction fields among the superparamagnetic nanoparticles, or even the ferromagnetic nanoparticles if the interaction fields are comparable to the coercivity. An interesting comparative analysis on this was given by Zamani Kouhpanji et al., where the authors compared the results of the hysteresis loops, remanence curves, first-order reversal curves (FORC), and projection method to assess the reliability and validity range these techniques for magnetic characterizing of nanoparticles [107].

#### 4.2.7. Magneto-Optic Kerr Effect (MOKE) Microscopy

**Figure 14:**
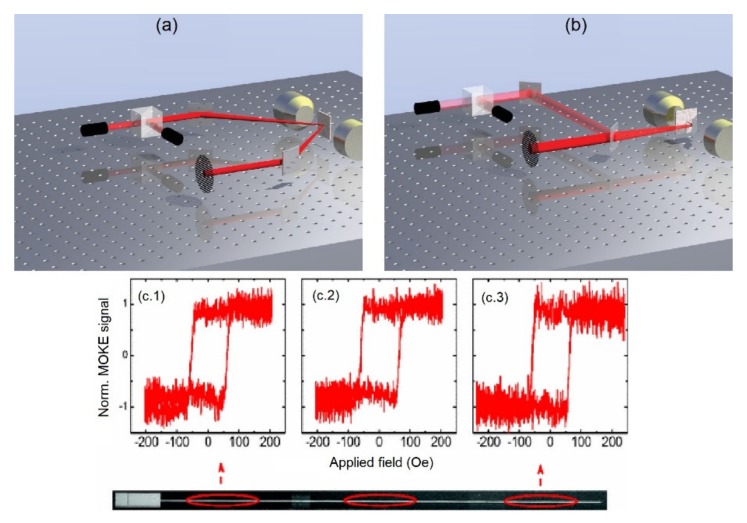
Schematic illustrating the magneto-optic Kerr effect (MOKE) setup [129]. (**a**) Out-of-plane measurement and (**b**) in-plane measurement, (**c.1**–**c.3**) show the MOKE signals at different locations along the magnetic nanowire corresponding to the red ellipses [132].

MOKE microscopy is an optical imaging technique that utilizes the interaction between the magnetization and optical waves to image the magnetization state on the surface of nanostructures (Figure 14). The MOKE measurements are categorized into four groups depending on the relative direction of the light polarization and the magnetization direction. The MOKE technique has been used to study the angular dependence of the magnetization demonstrating the anisotropy and the reversal mechanism of the magnetic nanoparticles [129]. For example, Palmero et al. reported on the coercivity mechanisms in multi-diameter magnetic nanowires illustrating the fundamentals for controlling the propagation of the single domain wall in the magnetic nanowires [130]. In another example, Bran et al. reported on capturing the Barkhausen jumps inside the magnetic/non-magnetic multi-segmented nanowires using the MOKE technique [131]. They showed that the magnetization can be pinned at the interface between the magnetic and non-magnetic segments that can be engineering using the ratio of the segments.

#### 4.2.8. Magnetic Force Microscopy (MFM)

The MFM technique is a form of scanning probe microscopy that measures the magnetic forces applied on the probe for imaging. In the MFM, a nanoscale probe is coated with a few tens of nanometers of magnetic material to capture the magnetic stray fields of the magnetic nanoparticles. The main advantage of the MFM is that it can image the magnetic domains indicating the magnetization direction. As an example, Mohammed reported on controlling the spin-torque driven domain wall motion in staggered magnetic nanowires using MFM [133]. Furthermore, it has been widely used to indicate the interaction fields among the magnetic nanoparticles [134]. Because of the high sensitivity of this technique, it has also been used to quantify the amount of magnetic nanoparticle internalized into the cells where optical imaging, such as fluorescence imaging is not feasible [135]. The MFM technique was originally proposed to measure the remanence magnetization in the absence of an external field, in other words, it used to only measure the remanence magnetization. However, recently several attempts were taken that enabled the whole hysteresis loop measurement using MFM in the presence of an external field. For example, Coisson et al. reported on implementing the MFM to probe the magnetic vortex chirality of magnetic nanodisc using the MFM technique [136].

#### 4.2.9. Minor Hysteresis Loops

Minor hysteresis loop measurements are performed at fields smaller than the saturation field of the magnetic nanoparticles. These measurements are very useful to describe the magnetization response of the magnetic nanoparticles that do not experience full saturation during the implementation of a cyclic magnetic field, such as during hyperthermia or nanowarming experiments. For example, Shore et al. conducted minor hysteresis loop measurements to demonstrate the heating efficiency of the magnetic nanowires for nanowarming applications [137]. Since the minor hysteresis loops are measured at not full saturation, the overall shape of the hysteresis loops highly depends on the initial magnetization state of the nanoparticle. As a result, the minor hysteresis loops technique has been used to study the reversal mechanism not only in magnetic nanoparticles but also in complex magnetic nanostructures.

#### 4.2.10. Zero-Field-Cooled/Field-Cooled (ZFC/FC) Magnetization Curves

**Figure 15:**
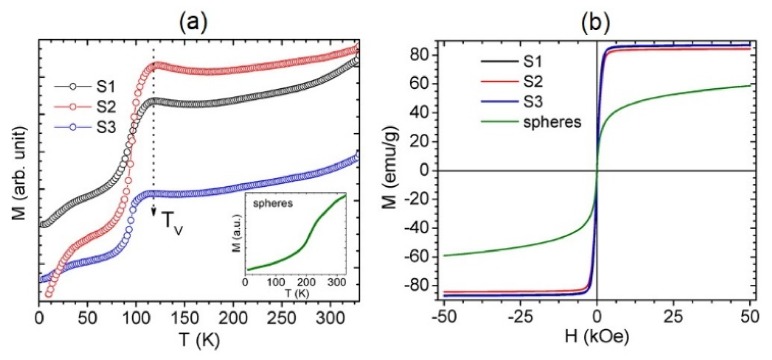
An example of the zero-field cooling (ZFC) measurement for three different types of superparamagnetic magnetic nanoparticles (**a**) and their corresponding hysteresis loops (**b**). As the temperature varies, there is a jump in the magnetization indicating the block temperature [140].

The ZFC/FC technique measures the magnetization at different temperatures to determine the transmission from ferri/ferromagnetic response to superparamagnetic response [138]. In ZFC, the nanoparticles are cooled to the lowest temperature in the absence of an applied field. Then, the magnetization is recorded at a finite field while the temperature increases (Figure 15). 

Initially, the magnetization increases with the temperature before it starts decreasing at a certain temperature, which is called the blocking temperature. The FC part of the measurement records the magnetization as the temperature is returned to the initial value. The blocking temperature has been widely used to determine the anisotropy of the magnetic nanoparticles. For example, Nemati et al. investigated the shape and size effects of nanospherical and nanocubic nanoparticles on their magnetic anisotropy and heating efficiency using the ZFC/FC technique [29]. In another example, Das et al. used the ZFC/FC technique as a probe for illustrating the structural transition of magnetic nanorods [139].

#### 4.2.11. Angular-Dependent Coercivity

The angular-dependent coercivity technique measures the hysteresis loop at different angles between the applied field and the easy axis of the magnetic nanowires. Intuitively, it is only applicable to magnetic nanoparticles having a strong shape anisotropy, such as magnetic nanowires or nanorods. The variation of the coercivity in terms of the angle determines the reversal mechanism in the nanoparticles, which can occur via a coherent rotation or a domain wall propagation [105]. It was shown that the reversal mechanism can changes from the coherent rotation to a domain wall at some angles, where there is a sharp reduction in the coercivity after an initial increase [141]. This technique also has been used to determine the energy barrier and magnetic anisotropy in nanoparticles [142]. In general, since this technique is unable to directly measure the interaction field and subtract its effects from the coercivity, this technique is not well-suited for characterizing the magnetic nanoparticles when the interaction fields are not negligible [143].

#### 4.2.12. First-Order Reversal Curve (FORC)

**Figure 16:**
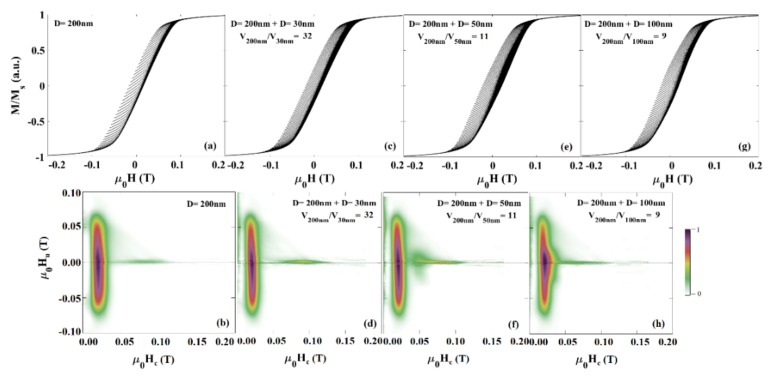
First-order reversal curve (FORC) data (**a**,**c**,**e**,**g**) and heat-maps (**b**,**d**,**f**,**h**) of magnetic nanowires in polycarbonate templates [143]. The horizontal and vertical axes of the heat-maps are the coercivity axes and interaction field axes, respectively.

The first-order reversal curve (FORC) technique is one of the most powerful techniques that has been widely utilized for quantitative and qualitative description of complex magnetic nanostructures [144]. Figure 16 provides an example of the FORC data and heat-maps for analyzing the types and volume ratios of magnetic nanowires combination. 

The FORC technique scans the whole area of the hysteresis loop leading to collecting detailed information regarding the interaction fields and intrinsic magnetic properties of the magnetic nanoparticles [145]. For example, Ramazani et al. utilized this technique to quantify the interaction fields and coercivity distribution of magnetic nanowire arrays [146]. In another example, Zamani Kouhpanji reported on the application of the FORC technique for demultiplexing the magnetic nanowires embedded inside the biological species [143]. Regardless of the complex data processing of the FORC [147,148,149], this technique is significantly slower than the other magnetic characterization techniques because it requires scanning the whole area of the hysteresis loop to a detailed analysis [107,108,129]. In this direction, several works have been done to enhance the measurements by introducing modifications to the standard protocol of the FORC technique. In a significant technological development, Zamani Kouhpanji et al. introduced a novel modification into the FORC protocol indicating only a narrow region of the hysteresis loop is required to fully characterize the magnetic nanoparticle assemblies [108]. They showed the interaction fields and coercivity of the magnetic nanoparticles can be comprehensively and reliably measured by a factor of at least 50×–100× faster than the standard FORC protocol [54,107].

#### 4.2.13. AC Field Technique

The AC field technique measures the hysteresis loops of magnetic nanoparticles at high frequencies. The AC field technique was first proposed to characterize the applied field rate effects on the coercivity, a critical parameter for proper designing magnetic recording media. This technique became a very important characterization for understanding the heating efficiency of the magnetic nanoparticles, especially the superparamagnetic nanoparticles because they have zero-coercivity at static field that increases with the frequency of the applied field. The coercivity of the superparamagnetic nanoparticles is linearly proportional to the frequency of the applied fields for low frequencies while it behaves nonlinearly at high frequencies. This frequency dependence of the coercivity of the superparamagnetic nanoparticles was found as a great source of heat generation for biomedical applications, such as hyperthermia [58,150,151] or nanowarming [152]. For example, Nemati et al. reported on the shape and size effects on the heat generation of magnetic nanoparticles using the AC field where it was shown the magnetic nanodiscs have relatively larger heating efficiency compared to the nanospherical nanoparticles [70]. Elsewhere, Das et al. reported on the enhanced heating efficiency of elongated magnetic nanoparticles using the AC field [139], where they showed the shape anisotropy induced by elongation is the key for tuning the heating efficiency of the magnetic nanoparticles. The requirement of a high heating generation rate for special applications, such as nanowarming of cryopreserved organs, the AC field technique became a strong technique for characterizing the magnetic nanowires, as they are capable to generate much higher heats granted to their shape anisotropy [137,153].

## 5. Applications

### 5.1. Drug Delivery

**Figure 17:**
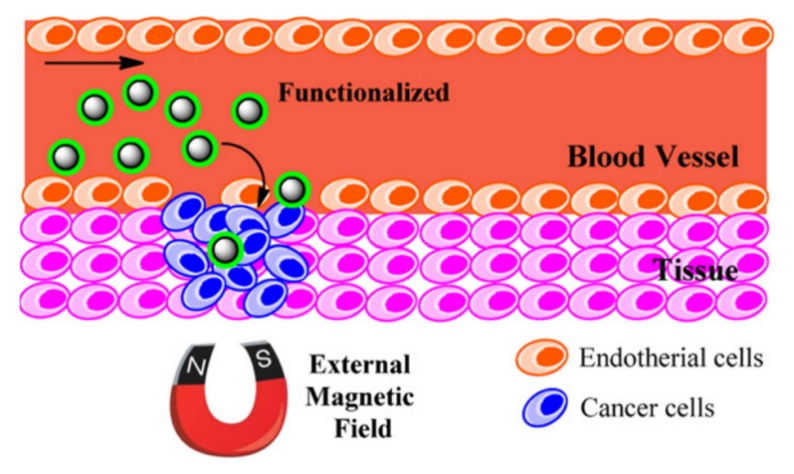
A schematic of magnetic nanoparticles for drug delivery. The magnetic nanoparticles concentrate at the targeted site using an external highly uniform and strong static magnetic field [30].

In traditional drug delivery approaches, either intravascular injection or oral ingestion, the blood circulation distributes the medication throughout the body. This drug delivery mechanism causes only a small portion of the medicine to reach the targeted tissue or organ while the majority of the drug is distributed to healthy sites. Due to the small therapeutic windows for effectiveness of a drug, this mechanism of drug delivery is not efficient as it requires injection of high doses to assure the effectiveness of the drug, which can cause side-effects on healthy tissues/organs. In this particular application, the magnetic nanoparticles are highly desired as they can be controlled and directed to the targeted sites using an external field [154] (Figure 17). The movement of the magnetic nanoparticles can be controlled using either a static magnetic field or an alternating magnetic field. Considering the costs and maneuvering flexibility, the static field is significantly cheaper but less accurate compared to the alternating field approach. Another advantage of magnetic nanoparticles in drug delivery is their capability for generating heat. In order to control the drug dissociation or activation, the loaded drugs on the magnetic nanoparticles will not be released unless temperature increases sufficiently to activate it [155]. As an example, Kennedy et al. reported in magnetic regulation of drug delivery using the ferrogels [156]. The authors showed that the stimulation of biphasic ferrogels using an optimized magnetic signal can control the drug release rates of multiple drugs at different frequencies.

Magnetic nanoparticles must have high magnetization and anisotropy to induce sufficiently large electromotive power to overcome the drag forces imposed by the blood. In this prospect, the elongated magnetic nanoparticles, especially the magnetic nanowires, have superiority over the other magnetic nanoparticles, such as nanospheres [157]. The magnetic nanowires with large magnetic moment and anisotropy were shown to penetrate deeply into the tissues targeting favorable sites. For example, Pondman and co-authors reported on non-toxic drug delivery using the magnetic nanowires that were surface functionalized in oleic acid [155]. In another study, Alsharif et al. proposed using the surface functionalized core-shell iron-iron oxide nanowire using BSA for drug delivery [158]. The larger magnetic moment of the core-shell iron-iron oxide improved the controlling the magnetic nanowires; however, due to the larger magnetic interactions, these magnetic nanowires show larger degrees of aggregation leading to reducing the drug delivery efficiency.

### 5.2. Cancer Therapy: Thermal or Mechanical

**Figure 18:**
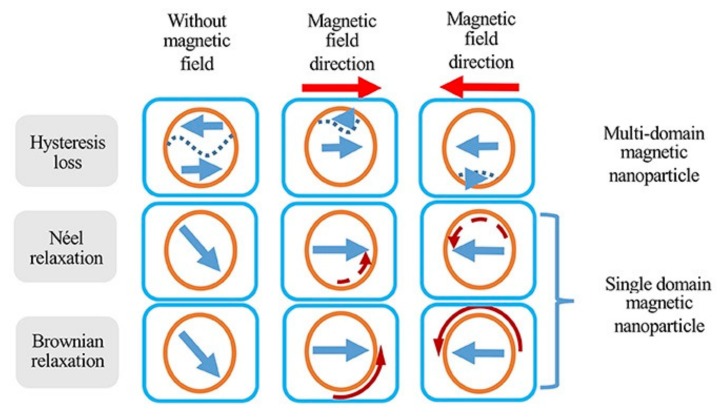
A schematic of the different heat generation modes using magnetic nanoparticles in cancer hyperthermia [161]. Orange circles represent magnetic nanoparticles, the straight arrows render magnetic field direction, curved arrows show the movement or change in magnetization direction (dashed curved arrow), and dashed lines illustrate domain boundaries in magnetic nanoparticles.

One of the most interesting applications of magnetic nanoparticles is cancer therapy [159]. Magnetic nanoparticles have shown great promise in destroying cancerous cells via both thermal damage and mechanical damage. In thermal damage, also known as hyperthermia, three mechanisms are used to destroy the cancer cells: (1) Brownian mechanism, (2) Neel mechanism, and (3) hysteresis loss (Figure 18). 

The Brownian mechanism damages the cancer cells by heat generation as a result of mechanical rotation and/or vibration, also known as external friction. The Neel mechanism damages cancer cells using the heat generation induced by the movement of the magnetic domains, which can be a result of the electromagnetic wave stimulus. The hysteresis loss, on the other hand, damages cancer cells using the heat generation induced by the switching the magnetic moment, as a result of an external magnetic field. Comparing the Neel mechanism and hysteresis loss, the hysteresis loss has significant advantage over the Neel mechanism as it can generate much higher heat rate. Comparing the Brownian mechanism and hysteresis loss, the hysteresis loss is practically more favorable because of its high selectivity for damaging the cancer cells with minimal damage to healthy ones compared to the Brownian mechanism [160].

In this particular application, the anisotropy and saturation magnetization of the nanoparticles play a critical role on their efficiency [150]. As a result, it was shown that the nanocubes, nanoflakes, nano-octahedra, elongated and nanodisc nanoparticles have significantly higher hyperthermia efficiency compared to nanospheres [162,163]. For example, Goiriena-Goikoetxea et al., reported on high yield synthesis of permalloy nanodiscs with zero remanence and high magnetic moment for cancer therapy [164], where the authors used the hole-mask colloidal lithography method to cost-effectively and uniformly produce nanodiscs. Larger hysteresis loss can be achieved by the nanoparticles with larger hysteresis area, such as electrodeposited ferromagnetic nanowires. For hysteresis loss, the external magnetic field must be sufficiently larger to meet the coercivity of the nanoparticles [77]. Since ferromagnetic nanowires have very large coercivities, they require a very large external field to generate heat. Superparamagnetic nanoparticles, on the other hand, have zero coercivity at static fields while it increases as the frequency of the external magnetic field increases [29]. Thus, even though the ferromagnetic nanowires have a significant higher heating efficiency, the superparamagnetic nanoparticles provide more flexibility for designing a more effective hyperthermia cancer therapy because the magnetic field and frequency cannot exceed the biological safety limits, as they can cause severe damage to the healthy cells/tissues.

### 5.3. Cryopreservation

Cryopreservation is another form of heat generation using magnetic nanoparticles; however, in contrast to the hyperthermia, it involves heat generation from cryogenic temperatures up to the melting temperature of the cryopreservation agent. In contrast to hyperthermia, the only heat generation mechanism is the hysteresis loss because the magnetic nanoparticles are not able to produce any mechanical rotation or vibration as they are fixed at cryogenic temperatures. The main challenge in this application is generating enough heat over a short time leading to a high heat generation rate. If the heat generation rate is not sufficiently large to meet the critical warming rate of the cryopreservation agent, large thermal stresses will form across the tissue/organ. These thermal stresses induce large the mechanical stresses that can cause mechanical cracks, and ultimately destroying the cryopreserved tissue/organ [165,166]. Spherical superparamagnetic nanoparticles have shown promising results as they meet the critical warming rate of VS55, a commonly used cryopreservation agent. Since VS55 is toxic, low concentrations are highly desirable to minimize its side-effects after transplanting. The lower concertation of the VS55 requires a higher heat generation rate that is very challenging to be met. In this case, other magnetic nanoparticles, especially ferromagnetic nanowires, are superb candidates [137] as they are able to generate heat at significantly higher rates. General speaking, since the ferromagnetic nanowires have large coercivities, they require large external magnetic field which makes their implementation problematic because at large magnetic fields the eddy currents cause extremely non-uniform heat distributions. This particular application of magnetic nanoparticles has been recently proposed and still much efforts are needed to elucidate the most effective nanoparticles, especially in the case of large cryopreserved tissues/organs that required very uniform temperature increase.

### 5.4. MRI Imaging

MRI images are captured using a strong magnetic field, magnetic field gradient, and radio waves. The main advantage of the MRI imaging is that this technique does not require X-rays or ionizing radiation compared to CT scans and PET scans. However, it is very challenging to take MRI images from tissues/organs with similar magnetic properties [165]. In these cases, magnetic nanoparticles have been used as contrast agents to distinguish those tissues/organs by manipulating the relaxivities, transverse (T2) and/or longitudinal (T1), of their molecules. The T1 and T2 relaxation times indicate the path that molecules/tissues return to their equilibrium state, transverse to the static magnetic field and parallel to the static magnetic field, respectively. The magnetic nanoparticles were found to be a promising candidate, especially for manipulating the T2 relaxation time that was found to be more effective compared to manipulating the T1 relaxation time, due to their non-zero magnetic moment. The most favorable T2 relaxation time can be achieved using the magnetic nanoparticles with highest local magnetic inhomogeneities. Thus, elongated nanoparticles, such as nanorods and nanowires, are good candidates for this application as they have higher magnetic inhomogeneity because of their high surface to volume ratio [167]. For example, Smolensky et al. reported a trend of increased T1 relaxation time with increasing the diameter of the nanospheres [168]. In another example, Zhou and co-authors reported on magnetic nanodiscs with enhanced T1 relaxation times compared to magnetic nanospheres [169]. In this direction, Shore et al. introduced Fe/Au multi-segmented magnetic nanowires where they observed the shorter segments with larger diameters have significant improved T2 relaxation time [167]. More recently, Martinez-Banderas et al. introduced core-shell iron-iron oxide magnetic nanowires with excellent T2 relaxation time suitable for cell tracking [170]. The authors varied the oxidization level of their electrodeposited Fe nanowires and they showed the oxide thickness can be adjusted to tailor the transverse relaxation time.

### 5.5. Tissue Engineering

**Figure 19:**
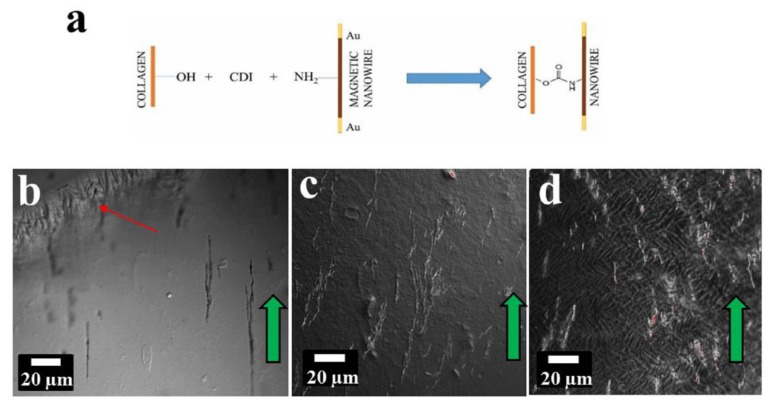
(**a**) A schematic of magnetic nanoparticles’ application for tissue engineering via crosslinking magnetic nanowires with collagen fibrils under a highly uniform magnetic field during gelation [176]; (**b**) a diffraction contrast image, (**c**) dark-field image, and (**d**) confocal reflectance image.

Tissue engineering is one of the major biomedical applications that has benefitted drastically from the capability of magnetic nanoparticles for being remotely actuated and/or controlled [171,172]. It was shown that engineering the texture of the tissues can successively improve nerve growth or cause cancer cell proliferation and migration to cease [173]. Traditionally, the fibrils of collagen-based tissues were oriented by applying a large magnetic field because the collagen is diamagnetic and it reorients perpendicular to the applied field direction [174]. Due to the requirement of very large magnetic field, in order of several thousand Oersted, this method is limited to a very small volume of the tissue. To overcome this limitation, Sharma et al. proposed crosslinking the collagen fibrils with surface functionalized magnetic nanowires prior to gelation. Using this technique, they showed that one-step bi-directional alignment of the collagen fibrils using only a few hundred Oersted [175] (Figure 19). 

Magnetic nanoparticles are also incorporated into hydrogels for producing ferrogels by chemically crosslinking the magnetic nanoparticles to the hydrogels. Ferrogel-based magnetic composites have attracted special attention, particularly in biosensing applications. An interesting work on the synthesis and comprehensive characterization of ferrogels was reported by Blyakhman et al. [177]. The authors synthesized and fully characterized two different series of ferrogels based on polyacrylamides with different chemical network densities. In another example, Safronov et al. reported on the synthesis of magnetically enriched polyacrylamide ferrogels by free radical polymerization using large aspect ratio magnetic nanowires, where they showed that large volume homogeneous magnetically enriched ferrogel-based tissues are feasible [5]. 

In addition to taking advantage of the remote actuation characteristics of magnetic nanoparticles for engineering regenerative tissues, magnetic nanoparticles also have been used in this particular application for inducing cells and delivering drugs promoting neuroregeneration. For example, Yuan et al. recently developed Au-coated superparamagnetic iron oxide nanoparticles to stimulate the nerve growth factor with low toxicity [176]. They showed that the coated magnetic nanoparticles provided higher neuronal growth and controlled orientation if they are stimulated under a dynamic magnetic field compared to a static magnetic field. Another interesting study was done by Polakka-Kanthikeel et al., where they showed that the surface functionalized magnetic nanoparticles are able to pass through the blood brain barrier to promote brain tissue repair [178]. Further information regarding the application and impacts of the magnetic nanoparticles in tissue engineering can be found in [173].

### 5.6. Enrichment

**Figure 20:**
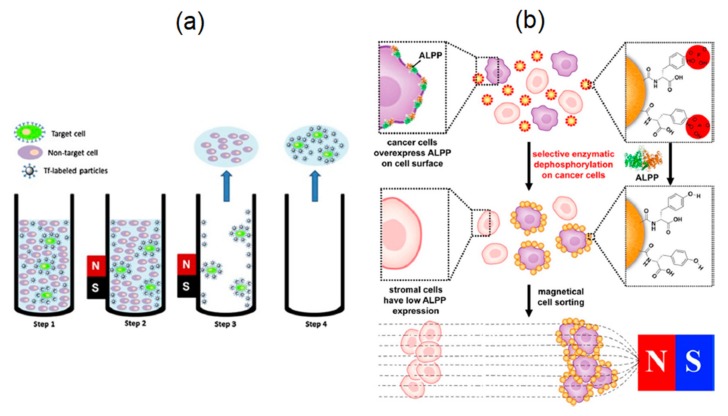
A schematic of the application of magnetic nanoparticles in enrichment [184]. (**a**) Scheme of cell separation using magnetic particles. Target cells are bound to magnetic particles modified with transferrin as a targeting moiety (step 1). Cells are magnetically separated (step 2). Non-targeted cells are removed with supernatant (step 3). Subsequently, target cells are re-suspended and removed (step 4). (**b**) Enzymatic transformation of magnetic particles for selective sorting of cancer cells.

Magnetic nanoparticles are promising enrichment agents compared to optical nanoparticles [2,179]. This is because the magnetically enriched biological entities, such as cells, can be rapidly and cost-effectively separated and isolated from the whole population using a simple magnet [160] (Figure 20). 

This can be readily done using a simple magnetic stand as opposed to costly, slow, and high complex optical systems. Magnetic nanospheres, nanocubes, and nano-octahedra have been widely used for this application because they can be mass produced cheaply. Furthermore, their rounded geometry, compared to the elongated nanoparticles, promotes their circulation inside capillaries with lower risk of blocking. However, there are two main challenges with these nanoparticles. First, they have relatively very small magnetic moment compared to ferromagnetic nanowires that makes it difficult to fully collect them. Second, ferromagnetic nanowires have shown a higher internalization yield that makes them superior for effective collect specific RNA/DNA and exosomes from targeted cells. For example, Hultgren et al. showed that nickel magnetic nanowires have an internalization yield of 35% larger than those of iron oxide nanospheres [180,181]. In another example, Sharma et al. observed high internalization of the gold-tipped nickel nanowires into osteosarcoma cells showing high cell activities and proliferation even after internalization of the magnetic nanowires [182,183]. In this direction, Nemati et al. enriched canine osteosarcoma cells (OSCA 8, 32, and 40) using iron-gold multi-segmented nanowires and they successfully collected the cancer-derived exosomes with a yield competitive to the commercialized methods [4].

### 5.7. Multiplexing/Demultiplexing Biolabels

Multiplexing/demultiplexing is crucial for many diseases, and tumors in particular, as they are often inhomogeneous. Multiplexing/demultiplexing can ensure both successful diagnosis and phenotyping of the discovered tumor [143]. Numerous attempts have been made to conjugate fluorophore molecules with magnetic nanoparticles to provide multiplexing. Since the multiplexing/demultiplexing of magnetic nanoparticles relies on the optical molecules, they inherently suffer the limitations of optical molecules [185]. The magnetic anisotropy of magnetic nanoparticles is a key for this application because it produces different magnetic signatures in different directions. Elongated magnetic nanoparticles, and in particular electrodeposited nanowires, exhibit a superior advantage over all other magnetic nanoparticles. That is because the electrodeposition technique provides much more flexibility to tailor the magnetic anisotropy via electrodepositing multi-segmented, multi-component, and multi-diameter magnetic nanowires with high-aspect ratios. For example, Sharma at al. electrodeposited multi-segmented magnetic nanowires for barcoding the cancer cells [183,184,186]. They used the hysteresis loop for detecting the barcodes, which suffers from low specificity [187]. Hysteresis loops only measure the saturation magnetization and the coercivity, which does not provide flexibility to generate multitude of unique detection. 

**Figure 21:**
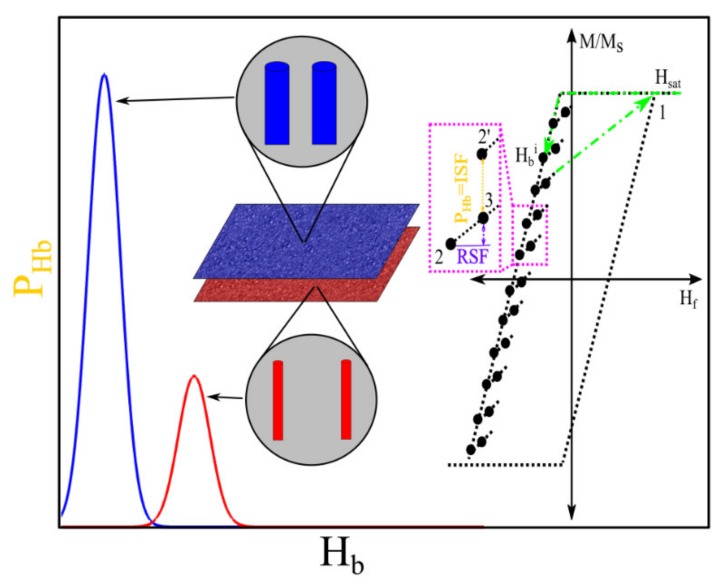
A schematic of the use of magnetic nanoparticles for multiplexing/demultiplexing [54]. The magnetic nanowires with different magnetic signatures, granted by their size and composition, were synthesized in polycarbonate tissues. The projection method, which directly measures the irreversible magnetization, was used to demultiplex the magnetically enriched tissues in complex combinations.

As an alternative, Wen et al. designed a ferromagnetic resonance system to detect the resonance frequency of the magnetic nanowires as biolabels [188]. The two main limitations of this approach are: (1) the magnetic nanowires must be in contact with a co-planar waveguide, a condition which cannot be met in practical applications, and (2) the magnetic nanowires must be aligned as their ferromagnetic signature changes in different directions. More recently, Zamani Kouhpanji et al. proposed an approach that suppresses the former limitations, as it can detect the magnetic nanowires regardless of its location in the biological tissue [143]. Using FORC measurements and proposing a novel approach for demultiplexing the overlap magnetic signatures, they were able to successfully quantify the magnetic nanowires with high precision competitive to optical techniques. In another significant technological development, Zamani Kouhpanji et al. introduced a novel approach to significantly speed up the measurement while collecting more accurate data than FORC technique to more precisely quantify the magnetic nanowires in complex combinations [54] (Figure 21).

### 5.8. Biosensing and Biosensors

**Figure 22:**
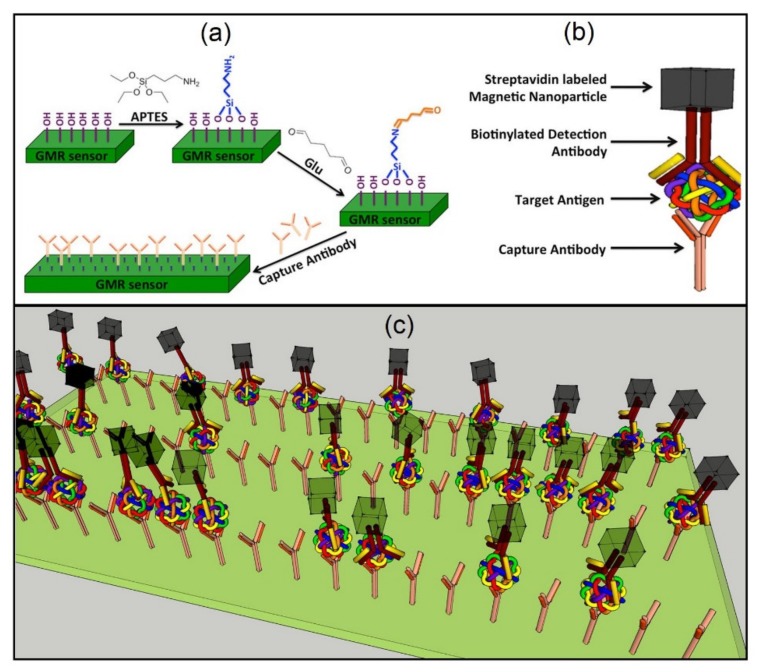
A schematic of the use of magnetic nanoparticles for biosensing applications [194]. In subfigures, (**a**) is a schematic diagram of a surface functionalized giant magnetoresistance (GMR) biosensor, (**b**) is the coated magnetic nanoparticles with a secondary antibody to detect the primary antibody on the surface of the GMR biosensor shown in (**c**).

Magnetic nanoparticles have broadly used in diverse nanosensors, especially biosensors [189]. The surface functionalization of the magnetic nanoparticles, such as aptamer-modified magnetic nanoparticles, is a critical key in biosensing as it not only enhances their biocompatibility but also their specificity [190]. The magnetic interaction between the magnetic nanoparticles is the basis of their application in biosensors granted to their large surface to volume ratio leading to very high binding affinity [191]. These interaction forces substantially decrease the time response of these nanosensors. In the majority of biosensors, the magnetic nanoparticles are suspended in a solution and there are surface functionalized to detect the target molecules (Figure 22). An early report of using the magnetic nanoparticles for sensing was by Wang at al. who utilized magnetic nanoparticles to detect DNA with high selectivity and sensitivity [192]. More recent studies, for example Tavallaie et al., reported on sensing the RNA with extremely low concentration, as low as 1 × 10^−17^M, using magnetic nanoparticles [193]. 

For this particular application, Maruyama et al. reported on a fully automated biosensors using biomagnetic nanoparticles [195]. The authors were able to detect and identify two types of somatic mutations of epidermal growth factor receptor gene in lung cancer in a limited time. Their results are interesting as they indicate a very low mutation rate could be detected using magnetic nanoparticles. The giant magnetoimpedance biosensors and giant magnetoresistance biosensors have been widely used for detection in biomedical applications [196]. A pioneering work on giant magnetoresistance biosensors was reported by Baselt et al. [197]. In this work, the authors were able to measure intermolecular force binding, such as DNA-DNA, ligand-receptor, and antibody-antigen, using their portable giant magnetoresistance biosensors. Blyakhman et al. developed a giant magnetoimpedance sensor prototype for studying ferrogels [176]. These forms of biosensors are very promising for dispersed magnetic nanoparticles in solutions or ferrogels. However, detecting the magnetic nanoparticles incorporated into living entities, such as cells or biological tissues, is extremely difficult and it still not properly understood.

## 6. Summary/Outlook

In this review, we have provided a short, but still comprehensive review, of the synthesis and characterization techniques of magnetic nanoparticles. The main focus of this review was to understand the most effective approach for synthesizing and characterizing magnetic nanoparticles suitable for specific bio-applications, particularly biosensing and nanomedicine. The sensitivity of a particular bio-application on the monodispersity or polydispersity of the magnetic property is the key to choosing the most efficient route for synthesis and characterizing the required magnetic nanoparticles. For example, in biosensing applications, the magnetic nanoparticles do not necessarily need to have an identical morphology. However, the final achievable magnetic moment and size, which determine the magnetic nanoparticles binding affinity, and costs for mass-production are the keys determining the synthesis technique. On the other hand, for a bio-application such as multiplexing where the identical magnetic property is in high demand, the synthesis technique must be capable of precisely tuning the magnetic properties. As another example, for drug delivery and tissue engineering applications it is critical to have magnetic nanoparticles with a strong magnetic moment to assure their controllability in addition to a large surface area to load a sufficient amount of drugs. In this particular application, the elongated magnetic nanoparticles, especially electrodeposited magnetic nanowires, are of great interest as they generally have a strong magnetic moment with a larger surface area. The same situation is valid for selecting the most appropriate characterization technique. For example, in hyperthermia cancer therapy and cryopreservation applications, the heating efficiency of the magnetic nanoparticles is the key that must be adequately characterized and verified using AC field, zero-field cooling/field cooling (ZFC/FC), and magnetic susceptibility measurements. Particularly for biosensing applications, the characterization techniques such as Fourier transform infrared spectroscopy (FTIR) or thermal gravimetric analysis (TGA) are very useful as they provide valuable information regarding the amount and types of the coatings. To date, even though the magnetic nanoparticles have been proposed as promising multimodal nanoparticles in diverse nanobiotechnology applications, there are still numerous broken connections among those that must be addressed to realize the multimodality of the magnetic nanoparticles.
